# Concurrent B Cell Acute Lymphoblastic Lymphoma/Leukemia and Monoclonal B Cell Lymphocytosis: A Case Report with Extensive Molecular Analysis

**DOI:** 10.1155/2022/1132544

**Published:** 2022-04-21

**Authors:** Maryam Mehdipour Dalivand, Parastou Tizro, Julie Partain, Anita Aggarwal, Jack Lichy, Francisco Mot Ara, Victor E. Nava

**Affiliations:** ^1^The George Washington University, Department of Pathology, Washington, DC, USA; ^2^Oregon Health and Science University, Portland, OR, USA; ^3^The George Washington University, Department of Medicine, Hematology and Oncology, Washington, DC, USA; ^4^Veterans Health Administration, Department of Internal Medicine, Oncology and Hematology, Washington, DC, USA; ^5^Veterans Health Administration, Department of Pathology, Washington, DC, USA; ^6^Hospital J.M. de Los Rios, Department of Pathology, Caracas, Venezuela

## Abstract

Although acute lymphoblastic leukemia (ALL) and monoclonal B cell lymphocytosis (MBL) are common neoplasia, a simultaneous presentation is very unusual. Here, we present two different B cell clones, MBL and B-ALL, cocirculating in a 78-year-old African American male. Detailed molecular characterization revealed an unusual MPL (T487I) point mutation and unmutated VH4-39. After nonstandard chemotherapy, the patient remains in morphologic remission. These findings may stimulate further research to clarify the pathogenesis of hematologic neoplasms.

## 1. Introduction

Acute lymphoblastic leukemia (ALL) is the second most common acute leukemia in adults [1] and the most common in children. The average lifetime risk of developing ALL is approximately 1 in 1000 and higher in white males older than 70 years with prior exposure to genotoxic stimuli [2].

This malignancy of unknown etiology has not been reported in association with monoclonal B cell lymphocytosis (MBL), which is defined as the presence of less than 5 × 10^9^/L clonal B cells in peripheral blood (PB) of asymptomatic patients without lymphadenopathy, organomegaly, or cytopenia, has been reported in up to 12% of the healthy individuals older than 40 years of age, and may be a precursor of chronic lymphocytic leukemia (CLL). MBL is divided into three subtypes: CLL-type (75% of cases) sharing immunophenotypic characteristics of CLL : CD19+, dim CD20+, CD5+, CD23+, dim surface immunoglobulin (Ig) light chain+, and FMC7−; atypical CLL-type: harboring atypical CLL immunophenotypic features, such as bright CD20, lack of CD23, and/or expression of FMC7; and non-CLL-type: CD5-negative and usually also lacking CD10 expression, as seen in marginal zone B cell lymphoma, lymphoplasmacytic lymphoma, and hairy cell leukemia. The epidemiology of MBL has strong parallels with CLL being associated with age older than 60 years, male gender, and family history of the disease. However, as with CLL or ALL, the exact etiology is unknown [3].

Here, we present a novel coexistence of B-ALL and CLL-type MBL with extensive molecular characterization.

## 2. Case Report

**Figure 1:**
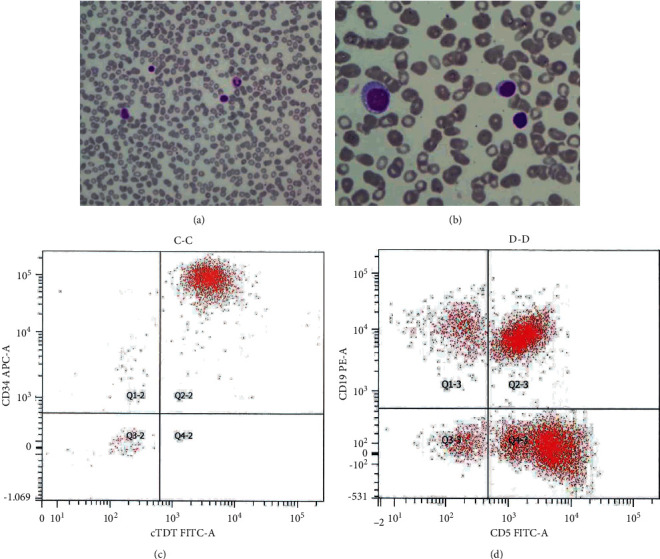
Peripheral blood smears (Wright-Giemsa stained) showing dimorphic lymphoid population at low magnification ((a), 400x) with a lymphoblast (arrow), two atypical small mature lymphocytes, and a band. (b) Lymphoblasts at higher magnification (1000x). Flow cytometric analysis of peripheral blood demonstrating B acute lymphoblastic leukemia with blasts coexpressing CD34 and TdT (c) and cocirculating monoclonal B cell lymphocytosis with aberrant coexpression of CD5 and CD23 (d).

A 78-year-old African American male with the medical history of Agent Orange exposure, hypertension, diabetes mellitus, benign prostatic hyperplasia, asthma, colon polyps, and sleep apnea presented to a primary care physician with intermittent back pain and fatigue for a month. A computed tomography scan revealed diffuse subtle mixed sclerotic/lytic bone lesions in the axial skeleton/iliac bones, which appear to be increasing in size over a period of 10 years. No relevant family history was identified. Clinical or radiological evidence of lymphadenopathy or organomegaly was absent. Multiple myeloma work up was negative (including normal serum and urine protein electrophoresis, serum kappa/lambda free light chains, beta2-microglobulin, and immunoparesis of IgA (<500 mg/L) and IgM (<50 mg/L)). However, an elevated lactate dehydrogenase (266 units/L) was noted. Complete blood counts showed normal white blood cell count (6700/mm^3^) with absolute lymphocytosis (4600/mm^3^, normal range 0.8–3.1/mm^3^), mild normocytic anemia (hemoglobin of 11.3 mg/dl), and normal platelets count (188000/mm^3^). PB smear review showed mild polychromasia, mild left shift (few myelocytes), few nucleated red blood cells, and atypical lymphocytosis comprised of two morphologically distinct populations (Figure 1). Most atypical lymphocytes represented mature small forms with subtle nuclear irregularities and clumpy chromatin, suggestive of low-grade lymphoproliferative disorder. However, a few undifferentiated blasts with fine chromatin, high nuclear: cytoplasmic ratio, and prominent nucleoli were also noted, concerning for high-grade hematopoietic malignancy (Figure 1).

**Figure 2:**
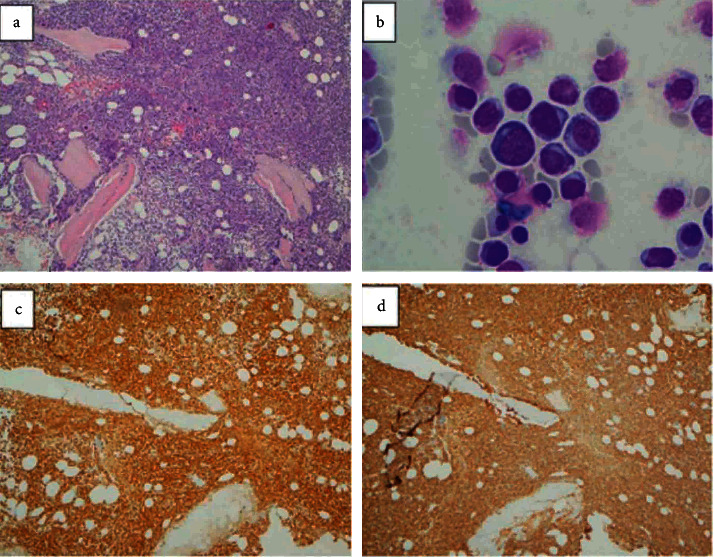
Bone marrow sections (hematoxylin and eosin stained) with sheets of lymphoblasts ((a), 100x). Heterogeneous lymphoblasts in bone marrow aspirates (Wright-Giemsa stained) at high magnification ((b), 1000x). Immunohistochemistry demonstrating positivity for TdT (c) and CD34 (d) on lymphoblasts in bone marrow sections (100x).

Flow cytometry (FC) analysis using standard CD45 vs. side scatter gating (Figure 1) revealed approximately 17% lymphoblasts positive for CD19, CD22, CD10, CD34, HLA-DR, and TdT and negative for CD20, CD5, CD23, CD25, CD103, CD11c, CD36, CD56, MPO, CD13, CD33, and CD14, compatible with evolving B-ALL. In addition, approximately 35% of lymphocytes (23% of total cells gated, 1600 lymphocytes/mm^3^) represented monoclonal surface kappa-restricted mature B cells (positive for CD19, CD20, CD5, CD23, CD25, and CD11c and negative for CD38, CD10, CD34, CD103, CD56, and FMC7), immunophenotypically consistent with CLL but at the level of monoclonal B cell lymphocytosis/monoclonal B cell lymphocytosis of uncertain significance (MBL/MLUS). Bone marrow (BM) examination confirmed the diagnosis of precursor B-ALL with approximately 76% lymphoblasts (Figure 2). Interestingly, only sparse reactive lymphoid aggregates composed by a mixture of CD3-positive T cells and approximately equal amounts of CD20 polyclonal B cells with no aberrant coexpression of CD5 or CD23 were identified by histopathology. However, FC analysis performed on BM also detected the MBL clone, which accounted for 8% of lymphocytes (8% of the total viable cells) and could represent a PB contaminant or more likely minimal involvement by MBL (below the level of detection by immunohistochemistry).

**Figure 3:**
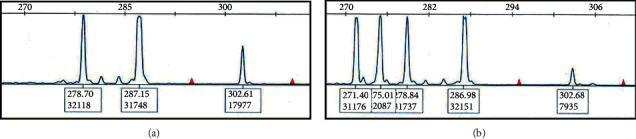
DNA isolated from the bone marrow aspirate and peripheral blood was analyzed for clonal rearrangements of the immunoglobulin heavy chain gene using the BIOMED-2 primer sets in the IGH Gene Clonality Assay Kit (InVivoScribe). Three PCR reactions were carried out with upstream primers in the FR1, FR2, and FR3 regions of the IGH gene and a downstream primer in the JH region. The images show results with the FR2-JH primer set.

In an attempt to confirm biclonality, DNA isolated from the PB specimen was analyzed by polymerase chain reaction (PCR) for clonal rearrangements of the immunoglobulin heavy chain (IgH) gene. This assay yielded five distinct PCR products with the FR1 and FR2 primer sets and four with FR3 (Figure 3). Only minimal polyclonal background was detected in an unsorted sample.

BM karyotyping of unstimulated cells revealed triclonal abnormal complexity, 46XY, add (20) (q11.2), add (21) (q1.1) [4], 47xy + 12 [3], and 46xy del (13) [2], in addition to normal background (46xy [5]). Clone 1 with an unbalanced rearrangement of 20q and 21q [4] was more compatible with acute leukemia or an underlying or evolving myelodysplastic syndrome/myeloproliferative neoplasm (MDS/MPN). In contrast, clone 2 (isolated trisomy of chromosome 12) and clone 3 (isolated deletion 13q) were more in keeping with a mature B cell lymphoid process, such as CLL. Fluorescent in situ hybridization (FISH) detected trisomy 10 and gain of an additional signal for RUNX1 at 21q22.12 (RUNX1/ETV6 probe) in 16% and 71% of cells examined, respectively. Additional FISH using a probe specific for 20q12 (DS0S108, Abbot Molecular) identified deletion of 20q12 in 57% of interphases, confirming the unbalance rearrangement of chromosome 20. No deletions of 6q, ATM (11q), 13q, or TP53 (17p) were found. These findings would indicate a neutral prognostic profile in CLL. Other aberrancies included in an ALL panel (BCR-ABL1 and KMT2A/MLL rearrangements) were not detected. Unexpectedly, DNA sequencing of IgVH revealed unmutated status in VH4-39, which has been associated with adverse prognosis, including transformation in CLL [5].

For further molecular characterization, we performed next generation sequencing (NGS) in the BM aspirate, using a custom designed Qiagen QIAseq v3 amplicon based targeted DNA comprehensive heme panel with genomic DNA run on an Illumina platform. Sequencing data were aligned against reference genome (hg19), and variants were called on an OHSU designed bioinformatics pipeline using multiple established variant callers (including FreeBayes, MuTect2, and Scalpel). The lower limit of detection for the assay is 2%.

A T487I point mutation was found in the thrombopoietin receptor gene **(**myeloproliferative leukemia protein/MPL/CD110), which is a known variant of uncertain significance according to ClinVar [4] and could represent a heterozygous germline variant, given the observed allelic frequency of 52%. Interestingly, this genetic alteration has not been reported in lymphoid neoplasms.

After obtaining cytologic confirmation that the cerebrospinal fluid was uninvolved by lymphoma, the patient was started on appropriate chemotherapy as per nonstandard B-ALL protocol. Because of his age, he was treated with MD Anderson B-ALL protocol with 1 cycle of hyperfractionated cyclophosphamide, vincristine, doxorubicin, dexamethasone (HCVD) and inotuzumab (Ino), mini-methotrexate, and cytarabine, followed by 4 cycles of consolidation and 4 cycles of maintenance with blinatumomab. Eight doses of prophylactic intrathecal methotrexate were also administered. The patient achieved remission, as demonstrated by BM and PB examination including FC analysis. Of note, the MBL clone was also undetectable. PB and BM marrow examination (including flow cytometry, IgH PCR, and karyotyping) demonstrated remission two months after treatment. However, BM cytogenetic analysis performed 1-year posttherapy demonstrated the original abnormal karyotype.

## 3. Discussion

CLL is the most frequent leukemia in adults, has been incidentally found in association with diverse solid tumors [6], and may be preceded by MBL. However, coexistence of CLL or MBL and other hematologic neoplasms is less frequent. Huang reported cocirculating B-ALL and CLL in an elderly woman [7].

Similarly, we report here for first time concurrent B-ALL and MBL with extensive molecular characterization.

Using PCR, at least four distinct peaks of IgH rearrangement were detected in PB which together with the observed dimorphic cell morphology strongly suggested a biclonal process. In addition, VH gene somatic mutation analysis revealed unmutated VH4-39 status, which has been reported in Richter's transformation [5] but not in association with acute leukemia.

NGS from a BM specimen identified a MPL (T487I) point mutation, which may be associated with congenital amegakaryocytic thrombocytopenia (CAMT), awaits further investigation in regard to ALL/MBL collision, and remains a VUS in ClinVar [4]. Various MPL mutations have been identified in myeloid neoplasms, including CAMT, hereditary thrombocythemia, MPN, refractory anemia with ringed sideroblasts associated with marked thrombocytosis, and acute myeloid leukemia [8]. Furthermore, a very similar MPL mutation (T487 A) was observed in a patient with acute megakaryoblastic leukemia, which induces a myeloproliferative disorder in mice through a cytokine-independent cell growth mechanism [9].

The MPL (T487I), we identified, has also been reported in nonhematopoietic malignancies (ovarian carcinoma and cervical pleomorphic liposarcoma) [10]; however, it is unreported in the unique setting of collision hematologic malignancy.

## 4. Conclusion

We present a novel coexistence of MBL and B-ALL with detailed molecular characterization, including a MPL (T487I) point mutation and VH4-39 unmutated usage, which may stimulate research to better understand the pathogenesis of hematologic neoplasms.

## Data Availability

The data used to support the findings of this study are available from the corresponding author upon request.
